# Involvement of Nrf2 in proteasome inhibition-mediated induction of ORP150 in thyroid cancer cells

**DOI:** 10.18632/oncotarget.6636

**Published:** 2015-12-18

**Authors:** Zhi-Hong Zong, Zhen-Xian Du, Hai-Yan Zhang, Chao Li, Ming-Xin An, Si Li, Han-Bing Yao, Hua-Qin Wang

**Affiliations:** ^1^ Department of Biochemistry & Molecular Biology, China Medical University, Shenyang 110001, China; ^2^ Key Laboratory of Cell Biology, Ministry of Public Health, and Key Laboratory of Medical Cell Biology, Ministry of Education, China Medical University, Shenyang 110001, China; ^3^ Department of Endocrinology & Metabolism, The 1st Affiliated Hospital, China Medical University, Shenyang 110001, China; ^4^ Department of Geriatrics, The 1st Affiliated Hospital, China Medical University, Shenyang 110001, China

**Keywords:** proteasome inhibitor, Nrf2, ORP150, thyroid cancer

## Abstract

Oxygen-regulated protein 150 (ORP150) is an inducible ER chaperone by numerous cellular insults and sustains cellular viability. We have previously reported that ORP150 is differentially induced in a panel thyroid cancer cells and represents as an unwanted molecular consequence during exposure to proteasome inhibition. However, the molecular basis for induction of ORP150 by proteasome inhibitors in thyroid cancer cells remains unclear. In the current study, we found that −421/−307 and −243/+53 regions at the *ORP150* gene were responsible for its transactivation by MG132 in thyroid cancer cells. Nrf2 directly transactivated the *ORP150* gene by direct binding with the −421/−307 region. Nrf2 also indirectly activated *OPR150* transcription via facilitating recruitment of ATF4 to the −243/+53 region. Collectively, this study highlights the molecular mechanism by which proteasome inhibition stimulates ORP150 expression via Nrf2 in thyroid cancer cells.

## INTRODUCTION

Proteasome inhibitors possess anti-tumor activity against hematologic malignancies and solid tumors [1]. Proteasome inhibition may lead to the accumulation of misfolded proteins in the endoplasmic reticulum (ER) lumen resulting in ER stress response, a process involving three ER transmembrane proteins: protein-kinase and site-specific endoribonuclease (IRE1), protein kinase R-like ER kinase (PERK), and activating transcription factor (ATF) 6 [5]. ER stressors induce phosphorylation of PERK and IRE1, which in turn lead to ATF4 activation and XBP1 splicing, respectively [16]. ER stressors also trigger cleavage of p90-ATF6 into p50-ATF6, which translocates to the nucleus and activates the target genes [32]. All ATF4, XBP1 and p50-ATF6 specifically activate transcription of ER stress response-related genes containing ER stress response element (ERSE) [17]. The ER-mediated apoptotic pathway is also triggered by ER stress, which leads to proapoptotic responses including induction of CHOP, activation of the apoptosis signal-regulating kinase 1 (ASK1)-c-Jun-N-terminal kinase (JNK) pathway and cleavage of ER resident caspases including caspase 12 (in rodent) and caspase 4 (in human) [11]. Accumulating studies now support that ER stress is implicated in the antitumor effects of proteasome inhibitors [7, 19, 23, 24, 30].

ORP150 is induced by various stimuli including ER stress, hypoxia, ischemia, glucose deprivation, reductive reagents, and functions as a survival mechanism [2, 4, 12–14, 18, 21, 25, 28]. ORP150 is also increased in various clinically isolated tumors and cancer cell lines [20, 27, 29]. We have previously shown that proteasome inhibition induces ORP150 expression in thyroid cancer cells [8]. ORP150 induction mediated by proteasome inhibitors is stronger in insensitive thyroid cancer cells than in those sensitive cells. In addition, suppression of ORP150 induction augments apoptotic cell death of thyroid cancer cells mediated by proteasome inhibitors [8], suggesting that ORP150 represents as an antiapoptotic factor during treatment with proteasome inhibitors. However, the mechanism(s) underlying preferential induction of ORP150 in insensitive thyroid cancer cells remains unclear. We have previously demonstrated that proteasome inhibitors primarily activate CHOP transcription via ATF4 binding to the *CHOP* promoter, while Nrf2 suppresses CHOP induction by precluding the recruitment of ATF4 to the *CHOP* promoter [33]. Since the competitive induction of CHOP and ORP150, in the current study, we explored the possible involvement of Nrf2 in induction of ORP150 by proteasome inhibitors in thyroid cancer cells.

## RESULTS

### Mapping the MG132-responsive elements at −243/+53 and −421/−307 region of the *ORP150* gene promoter in 8305C cells

**Figure 1:**
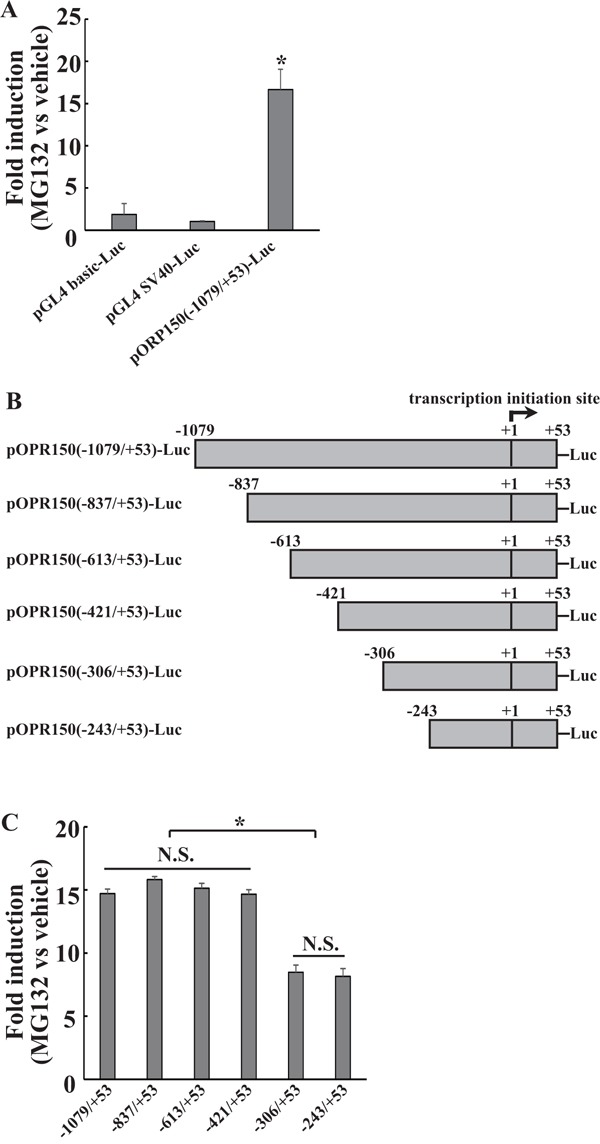
Mapping the MG132-responsive elements of *ORP150* gene at −243/+53 and −421/−307 regions **A.** 8305C cells were transfected with the indicated luciferase reporter constructs and pGL4.74[hRluc/TK] internal control plasmid. 24h after transfection, the cells were treated with vehicle or 2μM MG132 for additional 8h, then the luciferase activities were determined. **B.** Scheme of the truncated human *ORP150* gene promoter used in the current study. **C.** 8305C cells were transfected with one of the luciferase reporter constructs and pGL4.74[hRluc/TK] internal control plasmid. 24h after transfection, the cells were treated with vehicle or 2μM MG132 for additional 8h, then the luciferase activities were determined. *, *p*<0.05; N.S. not significant.

To examine whether transactivation of the *ORP150* gene might lead to its induction by MG132 in thyroid cancer 8305C cells, we used a reporter construct containing −1079 to +53 base pairs (bp) of the human *ORP150* promoter fused to luciferase (pORP150(−1079/+53)-Luc). 8305C cells were chosen for the current study, because they demonstrated the highest ORP150 induction by MG132 in a panel of thyroid cancer cell lines [8]. MG132 caused about 15-fold induction of the reporter gene activity in 8305C cell (Figure 1A). To further map the regulatory elements by MG132, a series of 5′ truncations of the *ORP150* promoter were constructed (Figure 1B). MG132 caused about 8-fold induction of pORP150(−243/+53)-Luc and pORP150(−306/+53)-Luc reporters (Figure 1C). pORP150(−421/+53)-Luc, pORP150(−613/+53)-Luc, pORP150(−837/+53)-Luc and pORP150(−1079/+53)-Luc reporters demonstrated about 15-fold induction upon MG132 exposure (Figure 1C). These data indicated that −243/+53 and −421/−307 regions of the *ORP150* gene might be responsible for its induction by MG132.

### Involvement of Nrf2 in transactivation of ORP150 by MG132 at both −421/−307 and −243/+53 regions

**Figure 2:**
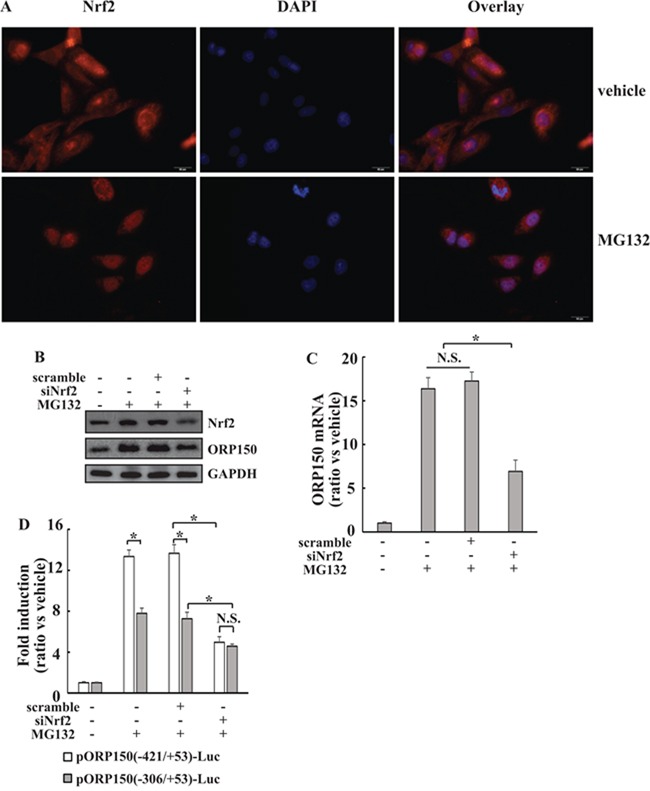
Implication of Nrf2 in *ORP150* induction by MG132 at both −421/−307 and −243/+53 regions **A.** 8305C cells were treated with vehicle or MG132 for 8h, nuclear localization of Nrf2 was observed using cell immunofluorescence. **B-C.** 8305C cells were transfected with siRNA against Nrf2 (siNrf2) for 48h, then treated with 2μM MG132 for another 8h, ORP150 protein (B) and mRNA (C) expression was analyzed using Western blot and real-time RT-PCR, respectively. **D.** 8305C cells were cotransfected with pORP150(−421/+53)-Luc or pOR150(−306/+53)-Luc reporter construct and siNrf2 for 48h, treated with MG132 for another 8h, then luciferase activities were measured. *, *p*<0.05; N.S., not significant.

In silico promoter analysis (http://www.sabiosciences.com) indicates that Nrf2 binding with the −340/−330 region of the *ORP150* gene promoter. In addition, we have previously reported that the Nrf2 expression levels are involved in the responsiveness of thyroid cancer cells to proteasome inhibition [6]. Therefore, we explored the potential involvement of Nrf2 in *ORP150* induction mediated by MG132 in 8305C cells. Immunofluorescence confirmed that 8305C cells inherently exhibited nuclear Nrf2 expression, and MG132 treatment stimulated its nuclear accumulation (Figure 2A). Specific siRNAs against Nrf2 (siNrf2) was then used to suppress the expression of Nrf2 in 8305C cells. siNrf2 successfully inhibited MG132-mediated accumulation of Nrf2, while scramble siRNA had no obvious effect (Figure 2B). Importantly, siNrf2 significantly blocked induction of ORP150 mRNA (Figure 2C) and protein (Figure 2B) expression induced by MG132. To investigate the regulatory site(s) of Nrf2, siNrf2 was then cotransfected with pORP150(−421/+53)-Luc and pORP150(−243/+53)-Luc, respectively. Compared with scramble siRNA, siNrf2 significantly decreased luciferase activities of both pORP150(−421/+53)-Luc and pORP150(−243/+53)-Luc reporters (Figure 2D). Notably, pORP150(−421/+53)-Luc and pORP150(−243/+53)-Luc reporters demonstrated similar transactivation activities in the presence of siNrf2 (Figure 2D), indicating that Nrf2 might be responsible for transactivation of the *ORP150* gene by MG132 at both −243/+53 and −421/−307 regions.

### Direct transactivation of ORP150 gene at −421/−307 region by Nrf2

**Figure 3:**
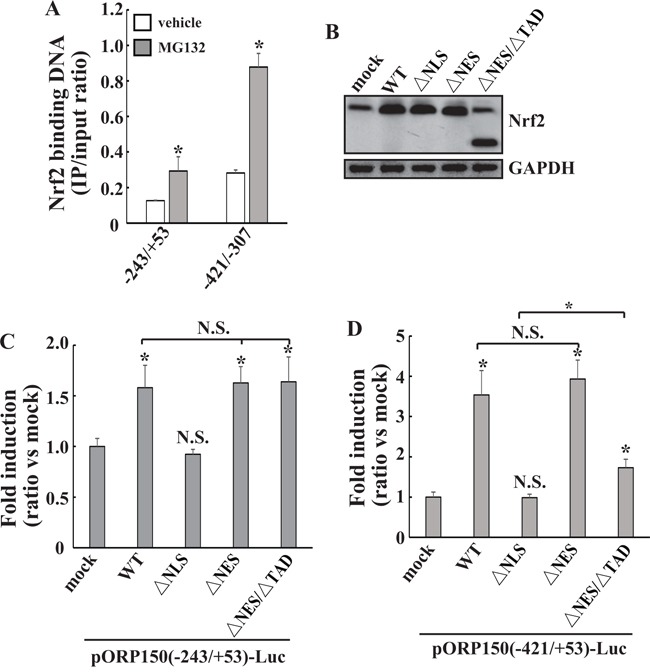
Direct transactivation of *ORP150* gene by Nrf2 at the −421/−307 region **A.** 8305C cells were treated with vehicle or 2μM MG132 for 8h, ChIP analysis was performed using an antibody against Nrf2, and immunoprecipitated DNA was amplified by real-time PCR. **B.** 8305C cells were transfected with mock or Nrf2 constructs for 24h, treated with 2μM MG132 for another 8h and Western blot was performed. **C-D.** 8305C cells were cotransfected with pORP150(−243/+53)-Luc (C) or pORP150(−421/+53)-Luc (D) reporter construct and one of the Nrf2 mutants for 24h, then treated with 2μM MG132 for additional 8h and luciferase activities were measured. *, *p*<0.05; N.S., not significant.

To investigate whether Nrf2 interacts with the −421/−307 and −243/+53 regions of *ORP150* promoter *in vivo*, ChIP analysis of 8305C cell extracts was performed using Nrf2 antibodies and PCR primers targeting the 115-bp and 297-bp DNA fragment, respectively. ChIP analysis showed that the fragments at both −420/−307 and −243/+53 regions of the *ORP150* promoter co-immunoprecipitated with Nrf2 antibodies, which was enhanced by MG132 exposure (Figure 3A), indicating that Nrf2 is associated with the upstream regulatory region of *ORP150*. To test whether Nrf2 directly activates the reporter genes, 8305C cells were cotransfected with various mutant Nrf2 constructs with pORP150(−243/+53)-Luc or pORP150(−421/+53)-Luc construct (Figure 3B). ΔNLS demonstrated no influence on the activity of both reporter genes under MG132 exposure (Figure 3C–3D), indicating that nuclear localization is critical for transactivation of ORP150 by Nrf2. WT, ΔNES and ΔNES/ΔTAD increased reporter activity of pORP150(−243/+53)-Luc with similar extent (Figure 3C), indicating that nuclear translocation was sufficient for transactivation of the *ORP150* gene at −243/+53 region. However, when compared with WT and ΔNES, ΔNES/ΔTAD mutant increased the reporter activity of pORP150(−421/+53)-Luc with smaller extent (Figure 3D), indicating that transactivation capacity of Nrf2 is required for full activation of the reporter gene. Collectively, these data indicated that Nrf2 directly transactivated the *ORP150* gene at-421/-307 region, while indirectly transactivated the *ORP150* gene at −243/+53 region.

### Indirect transactivation of ORP150 at −243/+53 region by Nrf2 via promoting ATF4 recruitment

**Figure 4:**
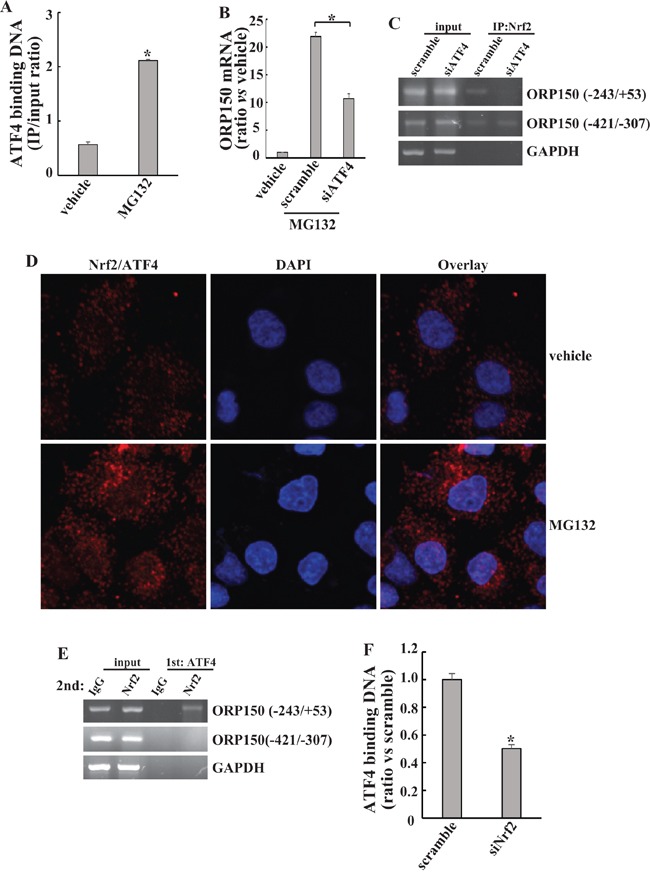
Indirect activation of *ORP150* at −243/+53 region by Nrf2 via interaction with ATF4 **A.** 8305C cells were treated with 2μM MG132 for 8h. ChIP analysis was performed using an anti-ATF4 antibody, immunoprecipitated DNA was analyzed using real-time PCR. **B.** 8305C cells were transfected with siRNA against ATF4 (siATF4) for 48h, then treated with 2μM MG132 for another 8h. ORP150 mRNA was analyzed using real-time RT-PCR. **C.** 8305C cells were transfected with scramble or siATF4 for 48h, then treated with 2μM MG132 for another 8h. ChIP analysis was performed using an anti-Nrf2 antibody, and immunoprecipitated DNA was analyzed using PCR and followed by gel electrophoresis. **D.** 8305C cells were treated with vehicle or 2μM MG132 for 8h, endogenous direct interaction of Nrf2 and ATF4 was analyzed using DuoLink PLA, and representative images were provided. **E.** 8305C cells were treated with 2μM MG132 for 8h, re-ChIP assay was performed to assess *in vivo* colocalization of Nrf2 and ATF4 to the ORP150 promoter. First ChIP and second ChIP antibodies were anti-ATF4 and anti-Nrf2, respectively. Immuoprecipitated DNA was analyzed using PCR and followed by gel electrophoresis. **F.** 8305C cells were transfected with scramble or siNrf2 for 48h, then treated with 2μM MG132 for another 8h. ChIP analysis was performed using an anti-ATF4 antibody, immunoprecipitated DNA was analyzed using real-time PCR. *, *p*<0.05; N.S., not significant.

The promoter of the *ORP150* gene contains an ER stress response element at −171/−179 region (ERSE) [10]. All ATF4, XBP1 and p50-ATF6 activate transcription of ERSE containing genes [17]. Since it has reported that Nrf2 interacts with ATF4 [28], we explored whether Nrf2 indirectly transactivated the *ORP150* gene at the −243/+53 region via interaction with ATF4. Firstly, ChIP analysis using an antibody against ATF4 demonstrated that MG132 increased recruitment of ATF4 to the −243/+53 region of the *ORP150* gene promoter in 8305C cells (Figure 4A). Transfection with specific siRNA against (siATF4) significantly suppressed MG132-mediated induction of ORP150 (Figure 4B). Knockdown of ATF4 by siATF4 almost completely blocked binding of Nrf2 to the −243/+53 region on the *ORP150* promoter in 8305C cells, while demonstrated no obvious influence on the binding of Nrf2 to the −421/−307 region (Figure 4C), indicating that binding of Nrf2 with −243/+53 region of the *ORP150* gene is indirect and ATF4 is indispensable for Nrf2 recruitment. Duolink PLA confirmed endogenous binding between ATF4 and Nrf2 in 8305C cells, which was enhanced by MG132 exposure (Figure 4D). In addition, re-ChIP by using ATF4 and Nrf2 antibodies sequentially demonstrated that after the ATF4 first ChIP enrichment of Nrf2 binding was observed in the *ORP150* promoter at −243/+53 region in 8305C cells (Figure 4E), indicating that Nrf2 and ATF4 were concurrently bound to the *ORP150* promoter at this region. On the contrary, Nrf2 binding was not observed in the *ORP150* promoter at −421/−307 region after the ATF4 first ChIP (Figure 4E). Importantly, Knockdown of Nrf2 expression by siNrf2 significantly decreased the binding of ATF4 to the ERSE element on the *ORP150* promoter in 8305C cells (Figure 4F), indicating that Nrf2 might stimulate the recruitment of ATF4 to the *ORP150* promoter.

### Positive correlation between ORP150 and Nrf2 in thyroid cancer tissues

**Figure 5:**
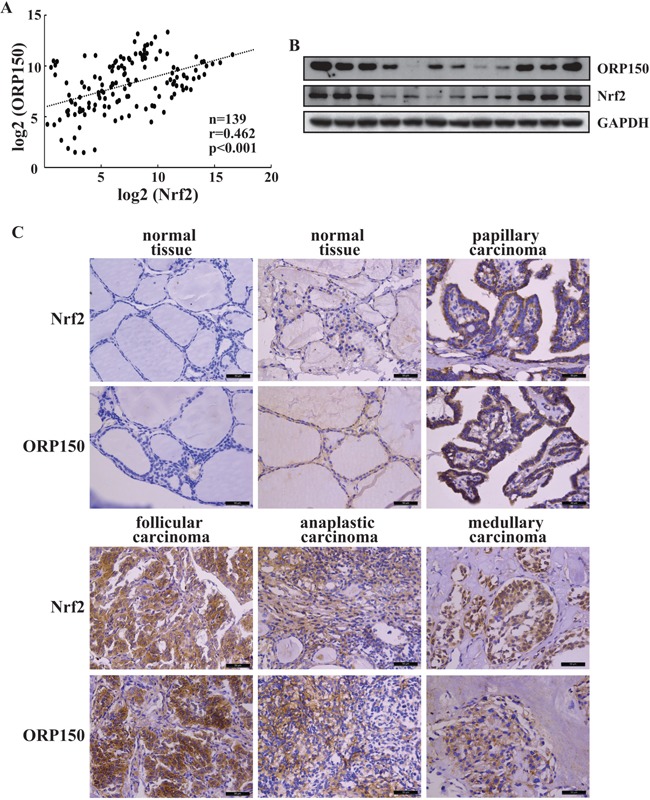
Positive correlation of Nrf2 and ORP150 expression in thyroid cancers **A.** ORP150 and Nrf2 mRNA levels were analyzed using real-time PCR and regression analysis was performed between the normalized ORP150 and Nrf2 mRNA levels in human thyroid cancer tissues. Each dot represents a sample, and the dotted line represents the linear regression fit, with the Pearson correlation coefficient (r) shown in the corner of the box. **B-C.** ORP150 and Nrf2 protein expression in human thyroid tissues were analyzed using Western blot (B) and immunohistochemistry (C), representative images were provided. Scale bar: 50μm.

Real time RT-PCR demonstrated the significant positive correlation between steady-state levels of ORP150 and Nrf2 in extracts from thyroid cancer tissues (Figure 5A). Consistent with mRNA expression levels, Western blot confirmed expression of Nrf2 and ORP150 proteins was positively correlated (Figure 5B). Immunohistochemistry demonstrated negative or relatively low signals of Nrf2 and ORP150 in normal thyroid tissues (Figure 5C). Nrf2 and ORP150 signals were clearly increased in 73 and 62 out of 109 analyzed carcinoma specimens, respectively (Figure 5C). In addition, most specimens demonstrated positive correlation of Nrf2 and ORP150 signals (Figure 5C), further supporting regulation of ORP150 by Nrf2 in thyroid cancer tissues.

## DISCUSSION

ORP150 is an inducible ER chaperone molecule that is increased by various stimuli including hypoxia, serum starvation, ischemia, ER stressors and proteasome inhibitors [2, 4, 8, 12–14, 18, 21, 25]. ORP150 is also increased to provide anti-apoptotic signals in a variety of cancers [3, 8, 20, 22, 25, 27, 29]. Although various ER stressors have been reported to induce ORP150, the underlying molecular mechanism has remained unclear. The only available information is that a 9-bp sequence located on the *ORP150* promoter region is almost identical to that of the ERSE with only 1-bp mismatch, to which p50-ATF6 specifically binds and activates its transcription [10].

The current study mapped two regulatory regions located at-421/-307 and −243/+53 of the *ORP150* promoter responsible for its induction by MG132 in 8305C cells. In silico promoter analysis demonstrated that one ERSE and a potential Nrf2 binding site located at −197/−191 and −340/−330 of the *ORP150* promoter, respectively. The current study demonstrated that Nrf2 was responsible for ORP150 induction at both −421/−307 and −243/+53 regions of its gene. Knockdown of Nrf2 expression decreased, while forced overexpression of Nrf2 enhanced ORP150 induction in 8305C cells. These data indicated that Nrf2 positively regulated ORP150 expression in thyroid cancer cells. The positive correlation between Nrf2 and ORP150 in human thyroid cancer tissues further confirmed regulation of ORP150 by Nrf2. Experiments using Nrf2 mutants demonstrated that Nrf2 directly activated ORP150 transcription at the −421/−307 region of the *ORP150* promoter. However, Nrf2 might not transcriptionally activate ORP150 directly, as Nrf2 mutant lack of transactivation domain increased the pORP150(−243/+53)-Luc reporter gene activity. ATF4 and Nrf2 concomitantly recruited to the −243/+53 region of the *ORP150* gene. In addition, knockdown of Nrf2 significantly decreased ATF4 recruitment to the-243/+53 region of the *ORP150* gene, indicating that Nrf2 might facilitate ATF4 recruitment to the *OPR150* promoter via direct interaction with ATF4.

**Figure 6:**
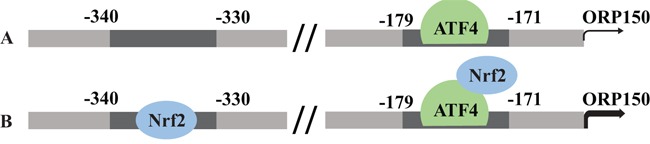
Model summarizing the proposed mechanism underlying differential OPR150 induction in thyroid cancer cells **A.** thyroid cancer cells with low Nrf2 expression. **B.** thyroid cancer cells with high Nrf2 expression.

Collectively, the current study demonstrated that Nrf2 positively regulated ORP150 expression in thyroid cancer. Nrf2 acts as a direct transcriptional activator at the −421/−307 region of *ORP 150* promoter, Nrf2 also facilitates the binding of ATF4 to the −243/+53 region of *ORP150* promoter (Figure 6). This report provides a comprehensive analysis of the regulation of ORP150 by Nrf2 in thyroid cancer.

## MATERIALS AND METHODS

### Culture of thyroid cancer cells

8305C cells were obtained from the European Collection of Animal Cell Cultures. The earliest passage of the cell line received in our laboratories was DNA profiled using the Applied Biosystems Profiler Plus kit (ABI, Foster, CA). Consistent with the previous report [26], the STR profile of the cell line was consistent with its profile in the DSMZ database (http://www.dsmz.de/). 8305C cells were maintained in DMEM (Sigma-Aldrich, Saint Louis, MO) supplemented with 10% fetal bovine serum (FBS, Sigma-Aldrich, Saint Louis, MO).

### Chemicals

MG132 was purchased from Calbiochem. 0.02% DMSO was used as vehicle control.

### RNA isolation and real-time reverse transcription-polymerase chain reaction (RT-PCR)

RNA isolation and real-time RT-PCR were performed as previously reported [30]. For ORP150, the forward primer was 5′-GTGCTGCAGCTCATCAATGAC-3′ and reverse was 5′-ATCTGCAGCTGTGGCTGCATC-3′, the amplicon size was 188 base pair (bp). For ATF4, the forward primer was 5′-TGACCTGGAAACCATGCCAG-3′ and 5′-AATGATCTGGAGTGGAGGAC-3′, the amplicon size was 221 bp. For β-actin, the forward primer was 5′-GAGACCTTCAACACCCCAGCC-3′ and the re erse was 5′-GGATCTTCATGAGGTAGTCAG-3′, the amplicon size was 205 bp. All the reactions were performed in triplicate, and the standard method was used for the quantification of the expression for each segment. The expression of targeted genes were expressed as arbitrary units, which was normalized by use of β-actin as a normalization control gene. All the reactions were done with a negative control to ensure that we had no contamination.

### Western blot analysis

Cells were lysed in lysis buffer (20 mM Tris-HCl, 150mM NaCl, 2mM EDTA, 1% Triton-X100 and protease inhibitor cocktail (Sigma-Aldrich, Saint Louis, MO). Cell extract protein amounts were quantified using the BCA protein assay kit. Equivalent amounts of protein (25μg) were separated using 12% SDS-PAGE and transferred to PVDF membrane (Millipore Corporation, Billerica, MA).

### Proximity ligation assay (PLA)

Duolink in situ proximity ligation assay was performed according to the manufacturer′s protocol (Sigma-Aldrich, Saint Louis, MO). PLA probes were diluted in 0.1% Triton X-100/PBS/1% fetal calf serum and incubated in a pre-heated humidity chamber for 1 h at 37°C, followed by hybridization, ligation, amplification, and detection. Fluorescence was visualized under a confocal microscopy (TCS SP5 Leica Microsystems).

### Small interfering RNA (siRNA)

The siRNA sequences used here were as follows: siRNA against Nrf2 (siNrf2), AAGAGUAUG AGCUGGAAAAAC; siRNA against ATF4 (siATF4), CCAGAUCAUUCCUUUAGUUUA. The scramble nonsense siRNA (scramble; CCGUAUCGUAAG CAGUACU) that has no homology to any known genes was used as control. Transfection of siRNA oligonucleotide was performed with Lipofectamine 2000 (Invitrogen, Carlsbad, CA ) according to the manufacturer′s recommendations.

### Chromosomal immunoprecipitation (ChIP) assay

ChIP analysis was performed as described previously [31]. Real-time quantitative PCR was performed using primers specific for human ORP150 sequence between −421 and −307 (forward: 5′-AAGCCTAGGGCACCTCA-3′ and reverse: 5′-TCTTCTTCCGGTCACCTG-3′), ORP150 sequence between −243/+53 (forward: 5′-CAGCAAAGCATCCAGCGC-3′ and reverse: 5′-GCG CGCTCATTGGAGCCTC). The immunoprecipitation/input ratio of the untreated sample was considered as 100% and the immunoprecipitation/input ratio of the MG132 treated sample was expressed as a percentage of the untreated.

### Construction of Nrf2 plasmids and cell transfection

A cDNA encoding human Nrf2 was generated by polymerase chain reaction (PCR) from human brain cDNA library (Invitrogen, Carlsbad, CA) and subcloned into the eukaryotic expression plasmid pcDNA3-Flag. Nrf2-ΔNLS (nuclear localization signal), Nrf2-ΔNES (nuclear export signal), and Nrf2-ΔNES/ΔTAD (transactivation domain) mutants were generated as previously reported [9]. The constructs were verified by DNA sequencing. Cells were transfected with Lipofectamine 2000 reagent (Invitrogen, Carlsbad, CA) as instructed by the supplier.

### Generation of *ORP150* promoter luciferase constructs and luciferase assay

The 5′-flanking region of human ORP150 genomic DNA between −1079 and +53 (+1 represents the transcription start site) was amplified by PCR from HEK293 genomic DNA and subcloned into the reporter plasmid pGL4 (Promega, Madison, WI) to generate pORP150(−1079/+53)-Luc. Serial truncated reporter genes were generated from pORP150(−1079/+53)-Luc by a polymerase chain reaction-based method. Cells were transfected with one of luciferase reporter constructs and pGL4.74 [hRluc/TK] (Renilla luciferase internal control) plasmid (Promega, Madison, WI). The firefly and renilla luciferase activities were determined using the Dual-Luciferase® Reporter Assay System (Promega, Madison, WI), according to the manufacturer′s instructions. All transfection experiments were repeated for three times in triplicate. The result was expressed as relative luciferase activity.

### Immunohistochemistry

Specimens from normal and pathological human thyroid tissues were isolated and immunohistochemistry was performed as previously reported [15].

### Statistics

The statistical significance of the difference was analyzed by ANOVA and post hoc Dunnett′s test. Statistical significance was defined as *p* < 0.05. All experiments were repeated three times, and data were expressed as the mean±SD (standard deviation) from three independent experiments.
